# A comparison of clinical features between idiopathic membranous nephropathy patients with and without serum antibody against phospholipase A2 receptor

**DOI:** 10.1097/MD.0000000000017658

**Published:** 2019-11-11

**Authors:** Qiuhua Zhang, Xiaobin Liu, Zhijian Zhang, Mian Wu, Biao Huang, Yi Zhang, Bin Liu, Zhen Qi, Weiwei Shan, Liang Wang, Zhigang Hu, Zhuxing Sun

**Affiliations:** a Wuxi People's Hospital Affiliated to Nanjing Medical University, Wuxi; b Nanjing Medical University, Nanjing; c School of Life Science, Zhejiang, Sci-Tech University, Hangzhou; d Jiangsu Institute of Nuclear Medicine, Wuxi, China.

**Keywords:** idiopathic membranous nephropathy, immunosuppressive therapy, phospholipase A2 receptor antibody

## Abstract

Patients with idiopathic membranous nephropathy (IMN) can be categorized into phospholipase A2 receptor (PLA2R)-associated and non-PLA2R-associated cases, according to serum PLA2R antibody status. The present study aimed to determine whether clinical features differed between these.

A total of 89 patients with IMN were retrospectively recruited for the present study. Serum PLA2R-Ab levels were determined by time-resolved fluoroimmunoassay. Furthermore, the relationship between serum PLA2R antibody levels and their responses to immunosuppressants among patients with a complete follow-up period, which was defined as at least 1 year, was analyzed.

Among these enrollees, 71 (80.0%) patients were positive for serum PLA2R antibody. Furthermore, patients with PLA2R-associated IMN had significantly higher age (with vs without, 54.31 ± 14.03 vs 46.67 ± 13.30 years old; *P* = .04), proteinuria (4.32 ± 1.84 vs 3.29 ± 1.90 g/d, *P* = .039), and serum albumin (25.33 ± 9.60 vs 31.38 ± 9.52 g/L, *P* = .019), but had lower serum immunoglobulin G (6.83 ± 2.89 vs 8.72 ± 2.95 g/L, *P* = .016) and erythrocyte sedimentation rate (47.31 ± 32.11 vs 26.33 ± 27.94, *P* = .013), when compared to IMN patients without PLA2R. Furthermore, IMN patients without PLA2R exhibited a better response to immunosuppressants, when compared to patients with PLA2R-associated IMN (without vs with, 66.7% vs 62.5% at 6 months and 100% vs 87.5% at 12 months), but the difference was not statistically significant.

Patients with PLA2R-associated IMN had higher disease severity than IMN patients without PLA2R. Furthermore, PLA2R negative patients had a better response to immunosuppressive therapies than PLA2R-positive patients, but the difference was not statistically significant.

## 1 Introduction

Membranous nephropathy (MN) is a common etiology of massive proteinuria. In China, the proportion of patients with MN has rapidly increased among patients with primary glomerulopathy, which ranged from 9.89% between 1979 and 2002 to 18.42% between 2003 and 2014.^[1]^ The main pathologic findings of MN include diffuse thickening of the glomerular basement membrane without features of hypercellularity, or subepithelial deposits of immunoglobulin G (IgG) and complement 3 (C3). Among all patients with MN, nearly 80% of these patients can be attributed to idiopathic MN (IMN), while the remaining patients are secondary MN (SMN)-related to diseases, including rheumatologic illnesses (such as systemic lupus erythematosus [SLE]), infections (such as hepatitis or syphilis), cancers, and medications.^[2]^ IMN can only be diagnosed after excluding other known etiologies based on clinical history, physical examinations, laboratory tests, and renal pathology.

However, the clinical features and prognosis of patients with IMN are highly variable. Furthermore, the spontaneous remission of nephrotic syndrome occurs in approximately 20% to 25% of patients with IMN, while approximately 40% of patients develop end-stage renal failure at 10 years after disease onset.^[3]^ The most recent Kidney Disease Improving Global Outcomes (KDIGO) guideline recommends the use of the severity of proteinuria as a marker of treatment responsiveness.^[4]^ However, the amount of proteinuria is not a sensitive indicator of treatment responsiveness or outcome in patients with IMN.^[5]^ Therefore, biomarkers that can accurately estimate disease activity in addition to proteinuria are urgently needed to monitor responses to any treatment, and predict clinical outcomes in patients with IMN.

In 2009, M-type phospholipase A2 receptor (PLA2R), a membrane glycoprotein localized to podocytes, was identified as a target antigen in patients with IMN.^[6]^ The prevalence of serum PLA2R antibody (PLA2R-Ab) varies from 57% to 82% among patients with IMN from different geographic areas.^[7]^ The discovery of serum PLA2R-Ab substantially contributes to the advancement of our understanding of the pathogenesis of MN. Serum PLA2R-Ab levels have been reported to correlate with disease activity, treatment response and long-term outcomes.^[8–11]^ However, the associations among serum PLA2R-Ab, clinical parameters, and remission rates in IMN patients remain to be investigated.

Different methods have been utilized to detect serum PLA2R-Ab, including enzyme-linked immunosorbent assay, western blot, and indirect immunofluorescence test. The investigators previously employed a highly sensitive time-resolved fluoroimmunoassay (TRFIA) to quantitatively detect serum PLA2R-Ab.^[12]^ In the present study, IMN was classified as PLA2R-associated and non-PLA2R-associated IMN based on serum PLA2R-Ab positivity, and the clinical images and treatment responsiveness between these 2 groups of patients were compared.

## 2 Materials and methods

### 2.1 Patient participation

In the present study, a total of 89 patients with biopsy-proven IMN were identified between January 2014 and December 2016. The present study was conducted at the Affiliated Wuxi People's Hospital of Nanjing Medical University in China. Patients with secondary forms of MN, including autoimmune diseases (lupus nephritis and Sjogren syndrome), infection-related MN (hepatitis B virus–associated MN, hepatitis C virus–associated MN, human immunodeficiency virus–associated MN, and syphilis), and MN correlated to malignancies or exposure to toxic agents, were excluded. Sera and urine were collected from the study participants, and none of these patients received immunosuppressants before inclusion. The anti-hypertension and anti-proteinuric medications of these patients were continued.

All study procedures were conducted according to the 2008 Declaration of Helsinki and good clinical practice guidelines. A written informed consent was obtained from each participant. The present study was approved by the Ethics Committee of the Affiliated Wuxi People's Hospital of Nanjing Medical University (no: Kyl2016001).

### 2.2 Clinical data

Serum creatinine (Scr), blood urea nitrogen, uric acid, albumin, total cholesterol, triglyceride, low-density lipoprotein cholesterol, high-density lipoprotein cholesterol, C3, complement 4 (C4), IgA, IgG, IgM, ESR, C-reactive protein, and hemoglobin were measured, and the estimated glomerular filtration rate was calculated using the Chronic Kidney Disease Epidemiology Collaboration equation. Urine was collected at the time of renal biopsy to assess the proteinuria amount using the average of 3 collections of 24-hour urinary protein and urine red blood cells. These patients were followed up for at least 1 year. The clinical data were recorded upon enrollment, and at 3, 6, and 12 months after immunosuppressant treatment. Patients were classified as having CR when they had proteinuria <0.3 g/d, and normal serum albumin and creatinine levels. Patients were classified as having PR when they had proteinuria <3.5 g/d and a 50% reduction in baseline levels, with improving serum albumin and creatinine, based on the KDIGO 2012 guidelines.

### 2.3 Renal pathologic examination

Light microscopy, immunofluorescence, and electron microscopy were harnessed to examine the renal biopsy specimens. Direct immunofluorescence for IgG, IgA, IgM, C3, C4, and C1q was performed on frozen tissue sections. These presented semi-quantitative results (0 to 4+). Furthermore, the dense deposits were also semi-quantitatively determined under electron microscopy (0 to 3+).

### 2.4 Time-resolved fluoroimmunoassay for PLA2R-Ab

Recombinant PLA2R was coated onto 96-well plates for the purpose of capturing the antibody. A goat-anti-human IgG tracer with europium-chelate was prepared for subsequent use. After bound/free separation by washing, the fluorescence counts of the bound tracer were measured to quantify the serum anti-PLA2R-IgG levels. A purified anti-PLA2R-IgG calibrator was 1st prepared to ensure that consistent results could be obtained. The detection range of anti-PLA2R-IgG based on TRFIA was 0.02 to 340.00 mg/L. The intra- and inter-assay coefficients of variation were 3.2% and 5.6%, respectively. The cut-off point for normal anti-PLA2R was set at <0.91 mg/L, according to existing literature.^[10,13]^

### 2.5 Statistical analysis

Statistical analysis was performed using the SPSS software version 18 (SPSS, Inc, Chicago, IL). Data were presented as mean ± standard deviation. *T* test was used for the parametric analysis. Categorical variables were presented in frequencies or percentages, and the data were analyzed by Fisher exact test. The differences were considered statistically significant when the *P*-value was <.05.

## 3 Results

### 3.1 Serum PLA2R-Ab measurement in patients with IMN

Eighty-nine patients with a pathologic diagnosis of IMN received tests for serum anti-PLA2R IgG. Among these patients, 71 (80.0%) patients had positive serum PLA2R-IgG (>0.91 mg/L).

### 3.2 Clinical baseline characteristics

The clinical features of the study participants at the time of renal biopsy are presented in Table 1. There was no difference in gender or Scr between IMN patients with and without PLA2R-Ab. However, patients with positive serum PLA2R-Ab significantly differed from patients with negative PLA2R-Ab in terms of age (with vs without, 54.31 ± 14.03 vs 46.67 ± 13.30 years old; *P* = .04), 24-hour proteinuria (4.32 ± 1.84 vs 3.29 ± 1.90 g/d, *P* = .039), and serum albumin (25.33 ± 9.60 vs 31.38 ± 9.52 g/L, *P* = .019). Furthermore, there were differences in serum IgG (6.83 ± 2.89 vs 8.72 ± 2.95 g/L, *P* = .016) and erythrocyte sedimentation rate (ESR; 47.31 ± 32.11 vs 26.33 ± 27.94, *P* = .013) between these 2 groups of patients.

**Table 1:**
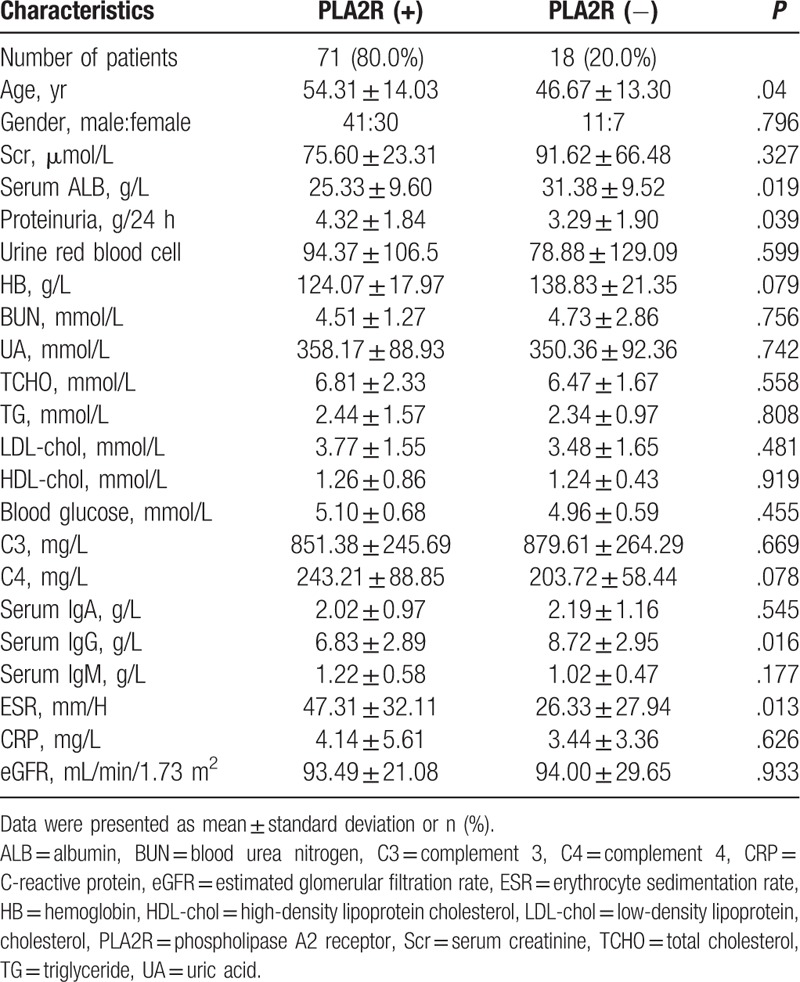
Clinical features of idiopathic membranous nephropathy patients with and without PLA2R-Ab at the time of renal biopsy.

### 3.3 Follow-up results at 12 months after renal biopsy

A total of 53 patients were followed up for longer than 12 months after renal biopsy. Among these patients, 42 and 11 patients had positive and negative serum PLA2R-Ab, respectively. Within the 1st year of renal biopsy, immunosuppressants were prescribed to 32 (76.2%) PLA2R-associated IMN patients and 11 (54.5%) IMN patients without PLA2R. Furthermore, cyclophosphamide (CTX) plus glucocorticoids were given to 22 PLA2R-positive and 5 PLA2R-negative patients, while tacrolimus plus glucocorticoids were given to 10 PLA2R-positive and 1 PLA2R-negative patient. The findings are summarized in Table 2.

**Table 2:**
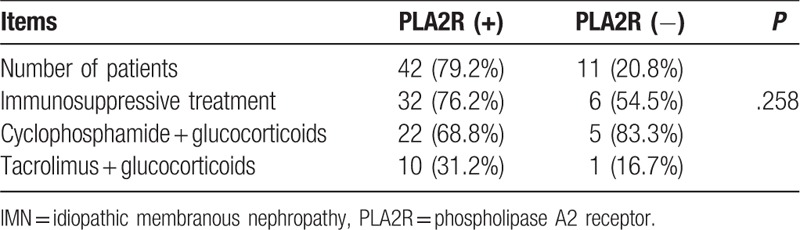
Treatment regimen for patients with PLA2R-associated and non-PLA2R-associated IMN during the 1st year of renal biopsy, n (%).

### 3.4 Therapeutic responses to immunosuppressive therapies

The complete remission (CR) and partial remission (PR) rates of proteinuria after 3, 6, and 12 months of immunosuppressive therapy were analyzed, and the results between IMN patients with and without PLA2R-Ab were compared. The immunosuppressive regimens comprised of 2 types: CTX and tacrolimus. The average proteinuric amount at the time of renal biopsy did not significantly differ between IMN patients (5.71 ± 1.51 vs 4.02 ± 2.02, *P* = .340), and between patients who used CTX and tacrolimus (CTX vs tacrolimus, 4.45 ± 1.57 vs 4.96 ± 1.64, *P* = .374).

The outcome data at 3 months after starting the immunosuppressive therapies were available in 32 PLA2R-associated and 6 non-PLA2R-associated IMN patients. The remission rates were higher in patients with positive serum PLA2R-Ab (CR: 9.4%, PR: 31.2%), when compared to IMN patients with negative PLA2R (CR: 0%, PR: 16.7%), but the difference was not statistically significant (*P* = .788). The findings are summarized in Table 3.

**Table 3:**
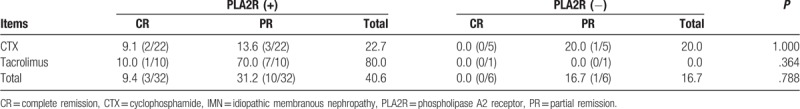
Treatment responses of patients with PLA2R-associated and non-PLA2R-associated IMN after 3 months of immunosuppressive therapy, % (n/N).

Next, the outcome data at 6 months after starting the immunosuppressive therapies in 32 PLA2R-associated and 6 non-PLA2R-associated IMN patients were analyzed. The remission rates (CR + PR) were higher in serum PLA2R-Ab-negative patients (66.7%) than in PLA2R-Ab-positive patients (62.5%), but the difference was not statistically significant (Table 4).

**Table 4:**
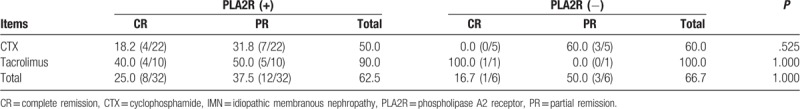
Treatment responses of patients with PLA2R-associated and non-PLA2RQ-associated IMN after 6 months of immunosuppressive therapy, % (n/N).

Finally, the outcome data at 12 months after starting the immunosuppressive therapies in 32 PLA2R-associated and 6 non-PLA2R-associated IMN patients were analyzed. The remission rates (CR + PR) were higher in serum PLA2R-Ab negative patients (100.0%) than in serum PLA2R-Ab positive patients (87.5%), but the difference was not statistically significant (*P* = .435, Table 5).

**Table 5:**
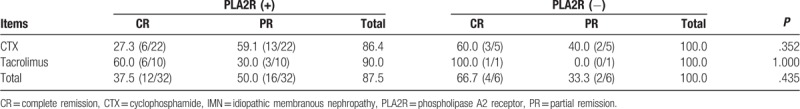
Treatment responses of patients with PLA2R-associated and non-PLA2R-associated IMN after 12 months of immunosuppressive therapy, % (n/N).

## 4 Discussion

By using TRFIA in the present retrospective, single-center cohort study, it was revealed that serum PLA2R-Ab was positive in 80.0% of patients with IMN. These present findings suggest that IMN patients with negative serum PLA2R had a significantly younger age, less proteinuria, and hypoalbuminemia, but had higher serum IgG, when compared to patients with positive serum PLA2R. In addition, IMN patients with negative PLA2R exhibited a better response to immunosuppressants after 6 and 12 months of immunosuppressive treatment. These observations improve our understanding of the clinical differences between IMN patients with and without PLA2R, and suggest that there may be potential differences in pathogenesis in these 2 groups of patients.

The PLA2R has been recognized as an important antigen in patients with IMN, and contributes to a deeper understanding of its pathogenesis. The in situ formation of the immune complex, which comprises of PLA2R and its antibody, activates the complement and generates the membrane attack complex, leading to sublethal podocyte injury, the disruption of the glomerular filtration barrier, and proteinuria. The detection of serum PLA2R-Ab facilitates the diagnosis,^[14]^ the monitoring of treatment responses,^[8]^ prognosis stratification,^[15]^ and the design of personalized therapies.^[16]^ The prior study conducted by the investigators and others have shown that the TRFIA-based quantification of serum PLA2R-Ab exhibits high accuracy in the diagnosis and prognostication of patients with IMN.^[10]^ Researchers have even recommended the replacement of renal biopsy with serum anti-PLA2R tests in IMN patients with relative contraindications to renal biopsy due to the high sensitivity and specificity of the anti-PLA2R assay.^[17]^

The present data reveals that patients with PLA2R-associated IMN had significantly lower serum IgG, but had higher age, ESR and proteinuria, and more severe hypoalbuminemia. Previous studies have demonstrated that glomerular PLA2R is an important antigen involved in the pathogenesis of IMN, and that serum PLA2R-Ab can effectively target PLA2R.^[3,18,19]^ These findings suggest that serum PLA2R-Ab is closely associated with the severity of proteinuria. In the present cohort, it was similarly found that patients with PLA2R-associated IMN had significantly more proteinuria and more severe hypoalbuminemia, but had less serum IgG, when compared to IMN patients without PLA2R. Since MN has been recognized as an organ-limited autoimmune disease, non-PLA2R-associated IMN may exhibit a resemblance to immune system disorders. It is likely that the presentations of the enrolled MN patients in the present study occurred at the early phase of certain systemic immune disorders, such as SLE. Alternatively, the negative status of PLA2R-Ab can result from the absence of disease activity at the time of blood sampling.^[18]^ A report supported the present proposition by showing that PLA2R-Ab levels measured at IMN diagnosis are inversely associated with the probability of achieving spontaneous remission.^[20]^ In the present study, the low proteinuria and higher serum IgG levels found in patients with non-PLA2R-associated IMN might represent the low clinical activity among these patients. Furthermore, the remission of proteinuria occurred in all non-PLA2R-associated IMN patients after 6 and 12 months of immunosuppressant use. These data suggest that non-PLA2R-associated IMN may exhibit lower clinical activity, although these results require further confirmation with the underlying mechanisms investigated. Part of the participants had an elevated ESR, which could be due to their occult infection at the time of blood sampling. There was no significant correlation between the IMN and ESR reported from existing literature.

Immunosuppressants, including corticosteroid alternating with alkylating agents and calcineurin inhibitors, are partially effective in reducing proteinuria in patients with MN. However, their use may be associated with the development of substantial adverse effects and relapse.^[21]^ The optimal immunosuppressive regimen for treating IMN remains controversial. Given the potentially different pathogenesis of PLA2R-associated and non-PLA2R-associated IMN, the investigators planned to determine whether these 2 groups of IMN patients responded differently to immunosuppressants. It was found that there was no difference in the proportion of patients who responded to immunosuppressants during the 1st year after biopsy between patients with PLA2R-associated and non-PLA2R-associated IMN in the present cohort. The treatment decision was made by the physicians based on the individualized plan tailored to the clinical presentations of these patients. These present findings indicate that clinical remission can be achieved in non-PLA2R-associated IMN patients after 6 and 12 months of immunosuppressant use. Previous reports support these present findings by showing that PLA2R-Ab negative IMN patients appear to have a higher spontaneous remission rate, when compared to PLA2R-Ab positive IMN patients, and immunosuppressant use did not alter the chance to achieve remission in PLA2R-negative patients.^[22]^ More studies are needed to determine the necessity and long-term efficacy of immunosuppressants in treating PLA2R-negative IMN patients.

The main limitation of the present retrospective study is the low sample size and relatively short follow-up duration. Hence, the interpretation of results correlated to long-term outcome, spontaneous remission and relapse rates can be difficult. Therefore, additional studies with a randomized prospective design and more enrolled patients should be conducted to validate the outcome associations found.

In conclusion, PLA2R-associated IMN patients have lower serum IgG, but have higher age and ESR, more proteinuria, and more severe hypoalbuminemia, when compared to patients with negative PLA2R-Ab. Furthermore, patients with non-PLA2R-associated IMN responded better to immunosuppressants, when compared to patients with PLA2R-associated IMN, although the difference was not statistically significant. Further studies are needed to determine the long-term clinical outcome in non-PLA2R-associated IMN patients.

## Author contributions

**Conceptualization:** Qiuhua Zhang, Biao Huang, Yi Zhang, Zhen Qi.

**Data curation:** Qiuhua Zhang, Xiaobin Liu, Weiwei Shan, Zhuxing Sun.

**Formal analysis:** Qiuhua Zhang, Xiaobin Liu, Biao Huang, Yi Zhang, Bin Liu.

**Funding acquisition:** Xiaobin Liu, Zhijian Zhang, Mian Wu, Bin Liu, Zhen Qi, Weiwei Shan, Zhigang Hu, Zhuxing Sun.

**Investigation:** Xiaobin Liu, Zhijian Zhang, Mian Wu, Biao Huang, Yi Zhang, Bin Liu, Weiwei Shan, Zhigang Hu.

**Methodology:** Zhijian Zhang, Mian Wu, Biao Huang, Bin Liu, Zhen Qi, Weiwei Shan, Liang Wang, Zhigang Hu.

**Project administration:** Zhijian Zhang, Mian Wu, Yi Zhang, Zhen Qi, Liang Wang, Zhigang Hu.

**Resources:** Liang Wang.

**Validation:** Liang Wang.

**Writing – review & editing:** Liang Wang.
